# Pre-treatment with GnRHa or *ulipristal acetate* prior to laparoscopic and laparotomic myomectomy: A systematic review and meta-analysis

**DOI:** 10.1371/journal.pone.0186158

**Published:** 2017-10-16

**Authors:** Inge de Milliano, Moniek Twisk, Johannes C. Ket, Judith A. Huirne, Wouter J. Hehenkamp

**Affiliations:** 1 Department of Obstetrics and Gynecology, VU Medical Center, Amsterdam, The Netherlands; 2 Department of Obstetrics and Gynecology, MC Zuiderzee, Lelystad, The Netherlands; 3 Medical Library, Vrije Universiteit, Amsterdam, The Netherlands; Seoul National University Bundang Hospital, REPUBLIC OF KOREA

## Abstract

**Background:**

Myomectomy has potential risks of complications. To reduce these risks, medical pre-treatment can be applied to reduce fibroid size and thereby potentially decrease intra-operative blood loss, the need for blood transfusion and emergency hysterectomy. The aim of this systematic review and meta-analysis is to study the effectiveness of medical pre-treatment with Gonadotropin-releasing hormone agonists (GnRHa) or ulipristal acetate prior to laparoscopic or laparotomic myomectomy on intra-operative and post-operative outcomes.

**Methods:**

We performed an extensive search in Embase.com, Wiley/Cochrane Library and PubMed in accordance with the Prisma guidelines. All studies published as full papers in peer reviewed journals using GnRHa or ulipristal acetate as medical pre-treatment independent of route of administration or dosage before laparotomic or laparoscopic myomectomy were included. The primary outcome was duration of surgery. Secondary outcomes were duration of enucleation, blood loss, degree of difficulty of surgery, identification of cleavage planes, proportion of vertical incisions, conversion rate, frequency of blood transfusions, post-operative complications, duration of hospital stay, frequency of recurrence of fibroids, frequency of uterine adhesions, recovery time and quality of life. No language restrictions were applied. Meta-analysis were performed where possible.

**Findings:**

Twenty-three studies were included. In laparotomic myomectomy, pre-treatment with GnRHa decreases intra-operative blood loss with 97.39ml (95% CI -111.80 to -82.97) compared to no pre-treatment or placebo. Pre-treatment with GnRHa before laparoscopic myomectomies also shows a reduction in intra-operative blood loss by 23.03ml (95% CI -40.79 to -5.27) and in the frequency of blood transfusions (OR 0.17, 95% CI 0.05 to 0.55) compared to no pre-treatment.

Only two retrospective cohort studies reported on pre-treatment with ulipristal acetate compared to no pre-treatment before laparoscopic myomectomy showing a statistically significant reduction in intra-operative blood loss, duration of surgery and frequency of blood transfusions after pre-treatment with ulipristal acetate.

**Conclusion:**

Administration of GnRHa prior to laparotomic myomectomy reduces blood loss and might decrease uterine adhesion formation. Pre-treatment with GnRHa before laparoscopic myomectomy reduces blood loss, the frequency of blood transfusions and might increase recurrence rate of fibroids, however it should be taken into account that some results are mainly based on cohort studies. Other pre-treatment agent ulipristal acetate has not been investigated sufficiently for relevant surgical outcomes.

## Introduction

Myomectomy is the surgical treatment option of choice for women who wish to preserve their fertility potential or wish to conserve their uterus for other reasons. A myomectomy can be performed by a laparotomy or a laparoscopy. Laparoscopic myomectomy has advan[^#tages over the laparotomic approach as the incisions are smaller and consequentially there is less post-operative pain, shorter hospital stay and faster recovery [1–3]. However, larger and numerous fibroids are more difficult to be excised by laparoscopy. The choice of surgical approach depends on number, size, location and type of fibroids and in particular the expertise of the surgeon and his team.

Myomectomy may be complicated by extensive peri-operative bleeding and as a consequence the need for blood transfusion and in some cases the need for an emergency hysterectomy. In the case of a laparoscopy, a conversion to laparotomy may be necessary. To reduce these risks, medical pre-treatment can help to reduce pre-operative bleeding, to increase pre-operative hemoglobin level, to reduce fibroid size and vascularization of the fibroid. Overall, pre-treatment can reduce peri-operative blood loss, the need for blood transfusion, emergency hysterectomy, conversion rates and may boost post-operative recovery and enable a quicker return to normal activities including work. Gonadotropin-releasing hormone agonists (GnRHa) are the most commonly used treatment that are supplied pre-operatively with the goal to reduce bleeding, fibroid size and its vascularity. After an initial temporary release of gonadotropins (i.e. flare-up), GnRHa induce a reversible suppression of gonadotropins by the desensitization and partly downregulation of pituitary GnRH receptors [4].

Recently an alternative therapy was launched for the pre-treatment of fibroid surgery. Ulipristal acetate is a selective progesterone receptor modulator that was recently been approved for pre-operative treatment of symptomatic fibroids. It reduces fibroid size due to its anti-proliferative, anti-fibrotic and pro-apoptotive effects on the fibroid [5]. It has similar effects on fibroid size reduction as GnRHa [6] and seems to have no effect on normal myometrium. To date, no other agents have been registered as pre-operative treatment of symptomatic fibroids prior to surgery.

The aim of this systematic review is to evaluate the effect of GnRHa or ulipristal acetate compared to no pre-treatment, placebo or any other medical pre-treatment prior to laparoscopic or laparotomic myomectomy on intra-operative and post-operative outcomes.

## Material and methods

We conducted a systematic review in accordance to the Prisma guidelines [7].

### Eligibility criteria

Studies fulfilling the following inclusion criteria were included:

#### Types of studies

We included prospective studies (including randomized controlled trials) and retrospective studies on pre-treatment prior to laparoscopic or laparotomic myomectomies. No language restrictions were applied.

#### Types of participants

Participants were pre-menopausal women of 18 years and older without no other underlying uterine pathology scheduled for a myomectomy (by laparoscopy or laparotomy).

#### Types of interventions

Admission of pre-treatment using GnRHa or ulipristal acetate versus no pre-treatment, placebo or any other medical therapy prior to surgery. No exclusion criteria for duration of pre-treatment were used.

#### Types of outcome measures

The following primary outcome was included:

- Duration of surgery (min)

Each of the following secondary outcomes were included:

1. **Intra-operative assessments:**
  - Duration of enucleation (min)
  - Blood loss intra-operative (ml)
  - Degree of difficulty of surgery
  - Identification of cleavage planes
  - Proportion of vertical incisions (in case of laparotomy)
  - Conversion rate (in case of laparoscopy)
2. **Post-operative assessments:**
  - Frequency of blood transfusions
  - Post-operative complications (e.g. fever, repeat surgery, pelvic hematomas)
  - Duration of hospital stay (days)
  - Frequency of recurrence of fibroids
  - Frequency of uterine adhesions (by second look laparoscopy)
  - Recovery time (return to normal activities and return to work)
  - Quality of Life (QoL) including Uterine Fibroid Symptoms and Quality of Life (UFS-QoL)

### Search methods for identification of studies

#### Electronic searches

A review protocol was developed based on the Preferred Reporting Items for Systematic Reviews and Meta-Analysis (PRISMA)-statement (www.prisma-statement.org). Embase.com, Wiley/Cochrane Library and PubMed were searched from inception (by IM and JK) up to 3 April 2017. The following terms were used (including synonyms and closely related words) as index terms or free-text words: ‘myoma’ or ‘myomectomy’ and ‘laparoscopy’ or ‘surgery’ and ‘ulipristal’ or ‘GNRH’ or ‘LHRH’. The full search strategies for all the databases can be found in the Supporting Information (S1 File). Duplicate articles were excluded. All languages were accepted.

### Data collection and analysis

#### Study selection

Two authors (IM and MT) independently assessed all potential studies for inclusion. If the title or abstract suggested presentation of a case eligible for inclusion, the full article was retrieved and reviewed against the inclusion criteria. Any disagreements were resolved by discussion. If no consensus was reached, a third author (WH) was asked to evaluate the full text of the study.

#### Data collection process

One reviewer (IM) extracted the data using a data extraction form which was developed by the authors, based on the Cochrane Consumers and communication Review Group’s data extraction template. A second reviewer (MT) checked the extracted data. Disagreements were discussed and when necessary a third reviewer (WH) was consulted. Authors were contacted in the case of missing data.

#### Quality assessment of the individual studies

The quality assessments were performed independently by two reviewers (IM and MT). For the included randomized controlled trials, the Cochrane Collaboration’s ‘Risk of Bias’ tool was used as described in the Cochrane Handbook [8]. Studies were classified as low risk of bias (+), unclear risk of bias (?) or high risk of bias (-). For all included non-randomized studies, the ‘Strengthening the Reporting of Observational Studies in Epidemiology’ (STROBE) checklist was used to assess the risk of bias [9]. We selected ten items of the checklist which were considered to be essential items for cohort studies. For the ‘methods’ section these items are: setting (item 5), participants (item 6), variables (item 7), data sources/ measurements (item 8), bias (item 9), study size/ power analysis (item 10) and statistical methods (item 12). For the ‘results’ section the following items were selected: participants (item 13), descriptive data (item 14), main results (item 16). Each item was scored as low (+), moderate (±) or high (-) risk of bias.

#### Data analysis

All analyses were performed separately for surgical approach initially chosen (laparoscopic or laparotomic) and type of study (randomized trial or cohort study). Mean fibroid volume before and after pre-treatment was presented descriptively in the study characteristics table. If only data for fibroid diameter (D) were available, fibroid volume was calculated applying the formula used for the volume of an ellipsoid: D1xD2xD3x0.5233. We performed meta-analysis using the fixed effect model if at least two studies reported on the following outcomes: duration of surgery, duration of enucleation, blood loss intra-operative, complication rate (including conversion rate, fever, repeat surgery), proportion of vertical incisions, frequency of blood transfusion, duration of hospital stay and recurrence of fibroids. A sensitivity analysis was performed for the primary outcome showing no differences in pooled results. All other outcomes were analyzed descriptively. For the meta-analysis, Review Manager (RevMan) version 5.3 was used. Where possible, data were pooled statistically by using mean and standard deviations (SD) and a forest plot was created. RevMan combines outcomes by calculating a weighted mean difference (WMD) and 95% confidence interval (CI). In case, means and SD were not directly available, these were derived from median and range using median as mean and estimating SD by ((range*0.95)/4)), or from mean, sample size, and standard error of the mean (SEM) where we computed the SD as SEM*(√n). If there was no range, SEM or SD reported, the article was excluded from the meta-analysis. If median and range was reported and range was judged too asymmetric around the median (in case of severe skewness), the article was also excluded from the meta-analysis. Heterogeneity (I^2^) of different studies was assessed by checking the results of the chi-squared tests. For dichotomous variables the pooled odds ratio (OR) with 95% confidence interval (CI) was calculated. In case of zero events in both treatment groups and no OR could be calculated, a sensitivity analysis was performed replacing 0 events by 1 event for both groups consecutive. Pooled OR and 95% CI were calculated. No further subgroup analyses were performed. Statistical significance was determined at p < 0.05.

## Results

**Fig 1:**
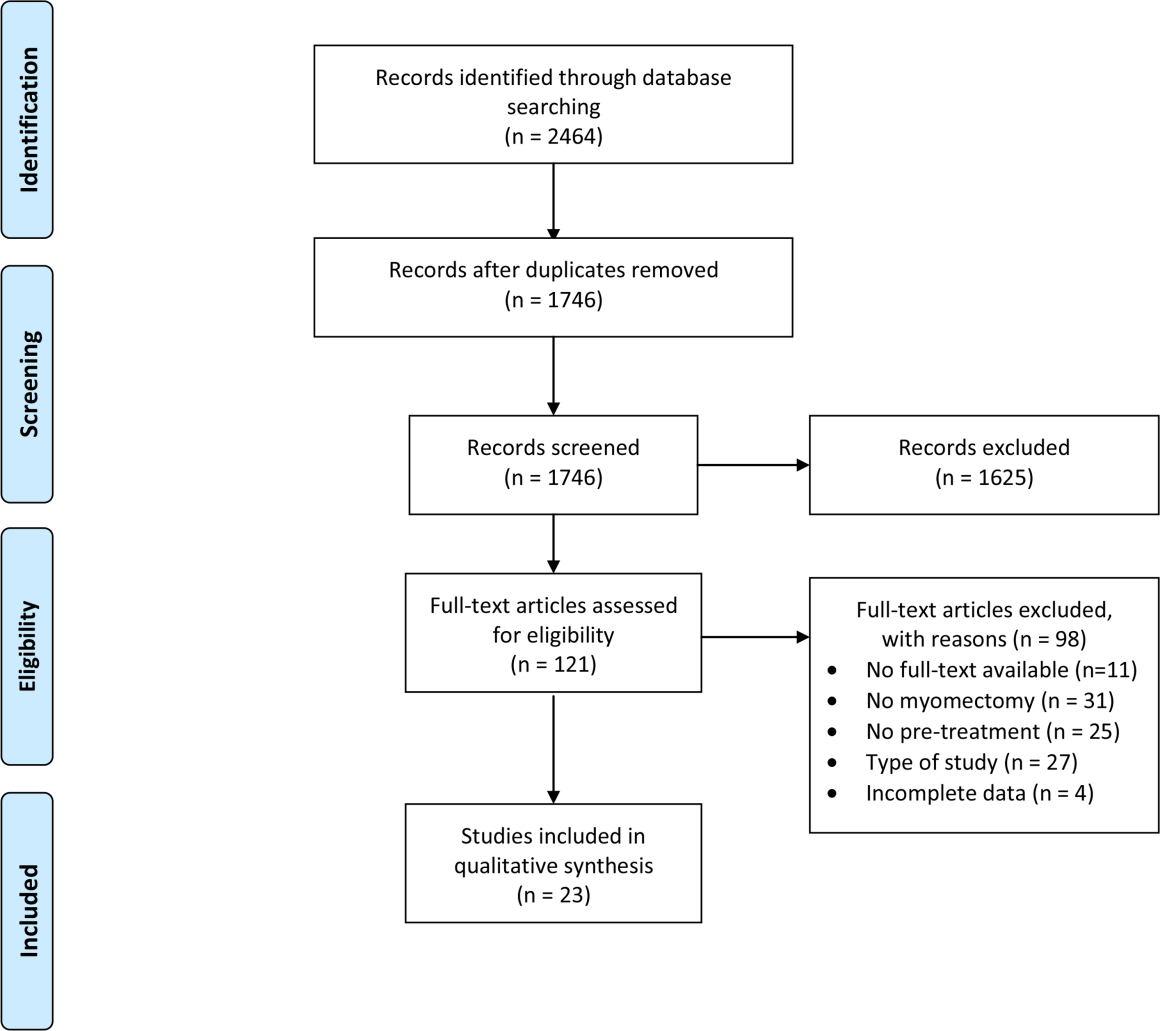
PRISMA Flow diagram. PRISMA (Preferred Reporting Items for Systematic Reviews and Meta-Analyses) flow diagram of study selection.

The search strategy yielded 2464 articles of which 718 were duplicates (Fig 1). In total 1625 articles were excluded based on title and abstract reading. A total of 121 references were considered potentially relevant and were selected for full-text reading of which 98 were excluded for various reasons (Fig 1). The authors of three studies were contacted, because hysterectomy and myomectomy data were not split and therefore data could not be used for this review [10–12]. One author was contacted due to insufficient data on type of surgery [13]. No reply was received and these four studies were all excluded for further data extraction and analysis. Finally, 23 studies met our inclusion criteria and were used for data extraction [14–36]. Fourteen of the included studies reported on laparotomic myomectomies, eight studies on laparoscopic myomectomies and one study reported on both laparotomic and laparoscopic myomectomies [22]. Twenty-one studies reported on GnRH agonists and two studies on ulipristal acetate. Study characteristics for all studies reporting on laparotomic myomectomy can be found in Table 1 and for the studies reporting on laparoscopic myomectomy in Table 2. Results will be presented separately for pre-treatment and surgical approach.

**Table 1:** Study characteristics (laparotomic myomectomies).

ID	Author, year	Blinded		Pre-treatment	Duration	No. of participants	Mean age(±SD)	Mean hemoglobine, g/dL (±SD)		Surgical approach	Use of vasoconstrictive medication peri-operative	Mean number of fibroids removed (±SD)	Mean fibroid volume, cm^3^ (±SD)	
		*Surgeons*	*Participants*					*Before pre-treatment*	*After pre-treatment*				*Before pre-treatment*	*After pre-treatment*
**Randomized clinical trials**														
**1**	Bustos Lopez, 1995 [14]	No	No	Nafarelin 200μg i.n.	Twice daily for 3 months	GnRHa: N = 13	GnRHa: 31.8±3.7	GnRHa: 13.2±2.4	GnRHa: 14.6±0.9	Pfannenstiel: N = 23	N.R.	N.R.	GnRHa: 29.2±23.5	GnRHa: 17.0±10.1
				vs. no pre-treatment		Control: N = 15	Control: 29.6±3.6	Control: 13.4±1.8		Vertical incision: N = 5			Control: 22.7±7.8	
**2**	Cetin, 1994 [15]	No	No	Buserelin 900μg i.n.	Daily for 3 months	GnRHa: N = 15	N.R.	GnRHa: 10.2±0.5	GnRHa: 13.4±0.6*	N.R.	N.R.	GnRHa: 3.2±2.9	GnRHa: 174.3±25.	GnRHa: 82.4±14.6
				vs. no pre-treatment		Control: N = 15		Control: N.R.				Control: 2.8±2.6	Control: 192.2±32.6	
**3**	Coddington, 2009 [16]	Yes	Yes	Leuprolide acetate 3,75mg	3 doses; interval monthly	GnRHa: N = 10	N.R.	N.R.	N.R.	Pfannenstiel	Vasopressin	GnRHa: 1.2±0.3	N.R.	
				vs. placebo		Placebo: N = 10						Placebo: 1.4±0.6		
**4**	De Falco, 2009 [17]	Yes	No	Triptorelin 3,75mg	3 doses; interval monthly	GnRHa: N = 33	GnRHa: 37.3±3.3	GnRHa: 9.9±1.3	GnRHa: 12.2±1.6*	Pfannenstiel	None	N.R.	GnRHa: 229.7±69.1	GnRHa: 118.8±53.1
				vs. no pre-treatment		Control: N = 29	Control: 37.5±3.1	Control: 9.7±1.5					Control: 229.7±65.5	
**5**	Fedele, 1990 [18]	No	No	Buserelin 1200 ug i.n.	Daily for 3 months	GnRHa: N = 8	33.6±3.3	N.R.	N.R.	N.R.	N.R.	GnRHa: 4.3±3.1	N.R.	
				vs. no pre-treatment		Control: N = 16						Control: 4.5±3.9		
**6**	Friedman, 1989 [19]	Yes	Yes	Leuprolide acetate 3.75mg	4 doses; interval monthly	GnRHa: N = 9	GnRHa: 35.2±1.1	GnRHa: 12.7±0.9	GnRHa: 12.2±1.2	Pfannenstiel: N = 16	Elastic tourniquet and vascular clamps	GnRHa: 6.0±5.4	N.R.	
				vs. placebo		Placebo: N = 9	Control: 34.9±1.1	Control: 12.9±0.9		Vertical incision: N = 2		Placebo: 2.6±1.8		
**7**	Friedman, 1992 [20]	Yes	Yes	Leuprolide acetate 3.75mg	4 doses; interval monthly	GnRHa: N = 9	GnRHa: 35.2±1.1	N.R.	N.R.	Pfannenstiel: N = 16	Elastic tourniquet and vascular clamps	GnRHa: 6.0±5.4	N.R.	
				vs. placebo		Placebo: N = 9	Control: 34.9±1.1			Vertical incision: N = 2		Placebo: 2.6±1.8		
**8**	Golan, 1993 [21]	No	No	Decapeptyl 3.2 mg i.m.	2 doses; interval monthly	GnRHa: N = 12	GnRHa: 36	GnRHa: 11.5±5.5	GnRHa:12.0±6.6	N.R.	N.R.	N.R.	N.R.	
				vs. no pre-treatment		Control: N = 9	Control: 38 (no SD reported)	Control: 11.0±4.8						
**9**	Hudecek, 2012 [22]	No	No	Goserelin acetate 3.6mg s.c.	3 doses; interval monthly	GnRHa: N = 78	33±5	N.R.	N.R.	N.R.	Methylergometrine and oxytocine i.v.	N.R.	N.R.	
				vs. no pre-treatment		Control: N = 44								
**10**	Imai, 2003 [23]	No	No	Buserelin 900 ug i.n.	Daily for 10–12 weeks	GnRHa: N = 10	GnRHa: 31.2±5.5	N.R.	N.R.	N.R.	Vascular clamps	GnRHa: 1.6±0.8	N.R.	
				vs. no pre-treatment		Control: N = 5	Control: 32.6±3.3					Control: 1.2±0.4		
**11**	Jasonni, 2001 [24]	No	No	Leuprolide acetate	6 doses; interval monthly	GnRHa 6m: N = 20	N.R.	N.R.	N.R.	N.R.	N.R.	N.R.	N.R.	
				vs. Leuprolide acetate	vs. 2 doses; interval monthly	GnRHa 2m: N = 16								
**12**	Vercellini, 2003 [25]	No	No	Triptoreline 3.75mg	2 doses; interval monthly	GnRHa: N = 49	GnRHa: 34±4	N.R.	GnRHa:12.7±1.2	N.R.	None	GnRHa: 3±3	N.R.	
				vs. no pre-treatment		Control: N = 48	Control: 33±4		Control: 12.3±1.1			Control: 3±3		
**Cohort studies**														
**13**	Bassaw, 2014 [26]	Yes	No	Goserelin 3.6mg s.c.	2 or 3 doses; interval monthly	GnRHa 2 doses: N = 18	GnRHa 2 doses: 34.2±5.6	N.R.	N.R.	Uterus >18–20 weeks gestation: vertical incision	None	N.R.	N.R.	
				vs. no pre-treatment		GnRHa 3 doses: N = 18	GnRHa 3 doses: 33.9±5.6			Uterus <18 weeks gestation: Pfannenstiel				
						Control: N = 32	Control: 35.0±4.9							
**14**	Falsetti, 1992 [27]	No	No	Goserelin depot 3.6mg	4 doses; interval monthly	GnRHa: N = 30	GnRHa: 32.2±3.1	GnRHa: 9.8±3.1	GnRHa:13.5±0.8*	N.R.	None	N.R.	N.R.	
				vs. no pre-treatment		Control: N = 35	Control: 35.5±2.3	Control: 12.4±1.0						
**15**	Kiltz, 1994 [28]	No	No	Leuprolide acetate 3.75mg	3 doses; interval monthly	GnRHa 3.75mg: N = 10	GnRHa 3.75mg: 28±1.3	N.R.	N.R.	N.R.	None	N.R.	N.R.	
				vs. Leuprolide acetate 7.5mg		GnRHa 7.5mg: N = 9	GnRHa 7.5mg: 30±2.1							
				vs. no pre-treatment		Control: N = 9	Control: 31±1.8							

*Statistically significant compared to baseline

**Table 2:** Study characteristics (laparoscopic myomectomies).

ID	Author, year	Blinded		Pre-treatment	Duration	No. of participants	Mean age (±SD)	Mean hemoglobine, g/dL (±SD)		Use of vasoconstrictive medication peri-operative	Mean number of fibroids removed (±SD)	Mean fibroid volume, cm^3^ (±SD)	
		*Surgeons*	*Participants*					*Before pre-treatment*	*After pre-treatment*			*Before pre-treatment*	*After pre-treatment*
**Randomized clinical trials**													
**1**	Campo, 1999 [29]	No	No	Decapeptyl 3,75mg i.m.	3 doses; interval monthly	GnRHa: N = 30	GnRHa: 34.9±5.1	N.R.	N.R.	In intramural fibroids >3cm: vasoconstrictor Glipressina	2.9±2.1	GnRHa: N.R.	GnRHa: 65.4±51.3
				vs. no pre-treatment		Control: N = 30	Control: 33.3±3.7					Control: 54.3±40.2	
**2**	Hudecek, 2012 [22]	No	No	Goserelin acetate 3.6mg s.c.	3 doses; interval monthly	GnRHa: N = 42	33±5	N.R.	N.R.	Methylergometrine and oxytocine i.v.	N.R.	N.R.	
				vs. no pre-treatment		Control: N = 48							
**3**	Palomba, 2001 [30]	No	No	Leuprolide acetate 3.75mg plus tibolone	2 doses; interval monthly	GnRHa+livial: N = 22	26.7±4.3	GnRHa+livial: 12.2±1.6	GnRHa+livial: 13.6±0.9*	Octapressin	N.R.	GnRHa+livial:179±48	GnRHa+livial:130±31*
				vs. Leuprolide acetate 3.75mg plus placebo plus iron tablets		GnRHa+placebo: N = 22		GnRHa+placebo: 11.9±1.5	GnRHa+placebo: 13.5±0.9*			GnRHa+placebo: 167±41	GnRHa+placebo: 113±23*
				vs. Only iron tablets		Control: N = 22		Control: 12.4±1.7	Control: 12.1±1.5			Control: 163±38	
**4**	Zullo, 1997 [31]	No	No	Leuprolide acetate 3.75mg	2 doses; interval monthly	GnRHa: N = 35	GnRHa: 36.8±4.1	GnRHa: 13.1±0.8	N.R.	Octapressine	GnRHa: 2.1±0.4	GnRHa: 62.8±28	GnRHa:41.5±24*
				vs. no pre-treatment		Control: N = 32	Control: 37.7±3.9	Control: 12.7±1.0			Control: 1.9±0.5	Control: 58.5±31	
**Cohort studies**													
**5**	Chang, 2015 [32]	No	No	Leuprolide acetate 3,75mg	3 doses; interval monthly	Leuprolide: N = 40	N.R.	N.R.	N.R.	Vasopressin	GnRHa: 3.4±3.3	GnRHa: 755.1±422.3	GnRHa: 321.4±184.2*
				vs. no pre-treatment		Control: N = 51					Control: 2.5±2.0	Control: 641.1±341.9	
**6**	Ferrero, 2016 [33]	No	No	Ulipristal acetate 5mg p.o.	Daily for 3 months	Ulipristal: N = 34	Ulipristal: 38.1±4.6	Ulipristal: 9.1±1.1	Ulipristal: 11.9±1.6	None	Ulipristal: 1.6±0.7	Ulipristal: 615.4±292.8	Ulipristal: 414.7±212.1*
				vs. no pre-treatment		Control: N = 43	Control: 37.5±4.0	Control: 9.2±1.2			Control: 1.5±0.7	Control: 583.3±405.5	
**7**	Luketic, 2017 [34]	Yes	No	Ulipristal acetate 5mg p.o.	Daily for at least 3 months	Ulipristal: N = 25	Ulipristal: 33.5±6.2	N.R.	N.R.	N.R.	Ulipristal: 1.1±0.3	Ulipristal: 203.6±111.7	N.R.
				vs. no pre-treatment		Control: N = 25	Control: 38.3±4.1				Control: 1.0±0.2	Control: 187.3±125.7	
**8**	Rossetti, 2001 [35]	No	No	GnRHa (dosage not reported)	N.R.	GnRHa: N = 24	36 (25–42)	N.R.	N.R.	Vasopressin	N.R.	N.R.	
				vs. no pre-treatment		Control: N = 54							
**9**	Sangha, 2016 [36]	No	No	GnRHa (dosage not reported)	N.R.	GnRHa: N = 15	N.R.	N.R.	N.R.	N.R.	N.R.	N.R.	
				vs. no pre-treatment		Control: N = 102							

*Statistically significant compared to baseline

### Risk of bias in included studies

**Fig 2:**
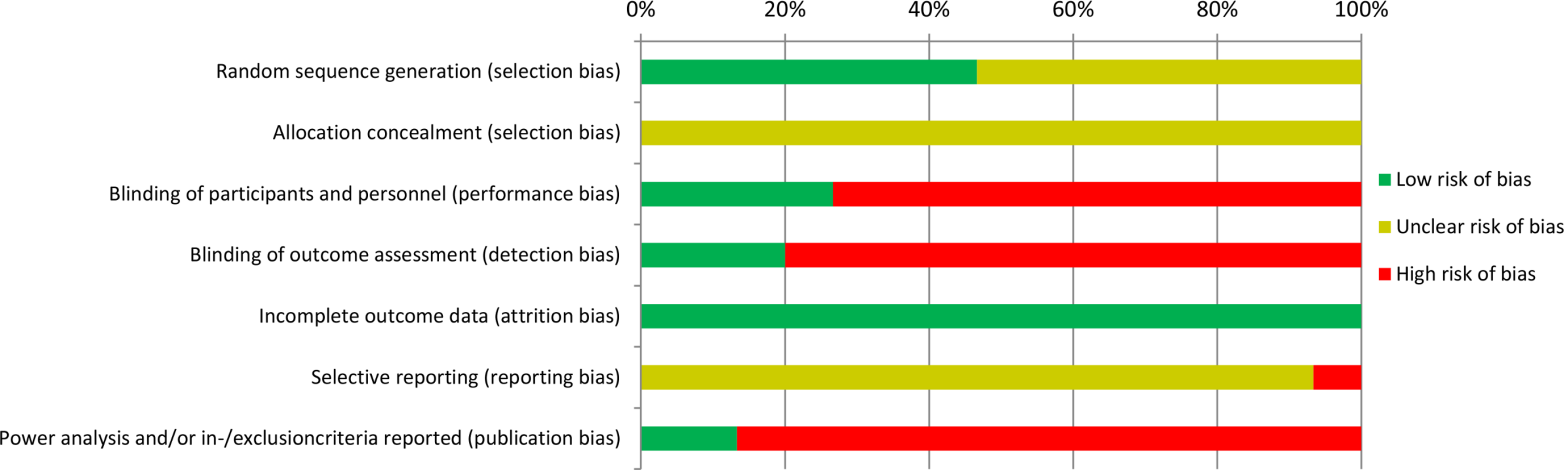
Overall risk of bias for included randomized controlled trials (RCT’s). ‘Risk of bias’ tool was used as described in the Cochrane Handbook. Studies were classified as low risk of bias (+), unclear risk of bias (?) or high risk of bias (-). Quality items: random sequence generation (selection bias), allocation concealment (selection bias), blinding of participants and personnel (performance bias), blinding of outcome assessment (detection bias), incomplete outcome data (attrition bias), selective reporting (reporting bias) and power analysis and/or in-/exclusioncriteria reported (publication bias). This figure shows the overall quality of included RCT’s qualified by low risk of bias (green colored bar), unclear risk of bias (yellow colored bar) and high risk of bias (red colored bar).

**Fig 3:**
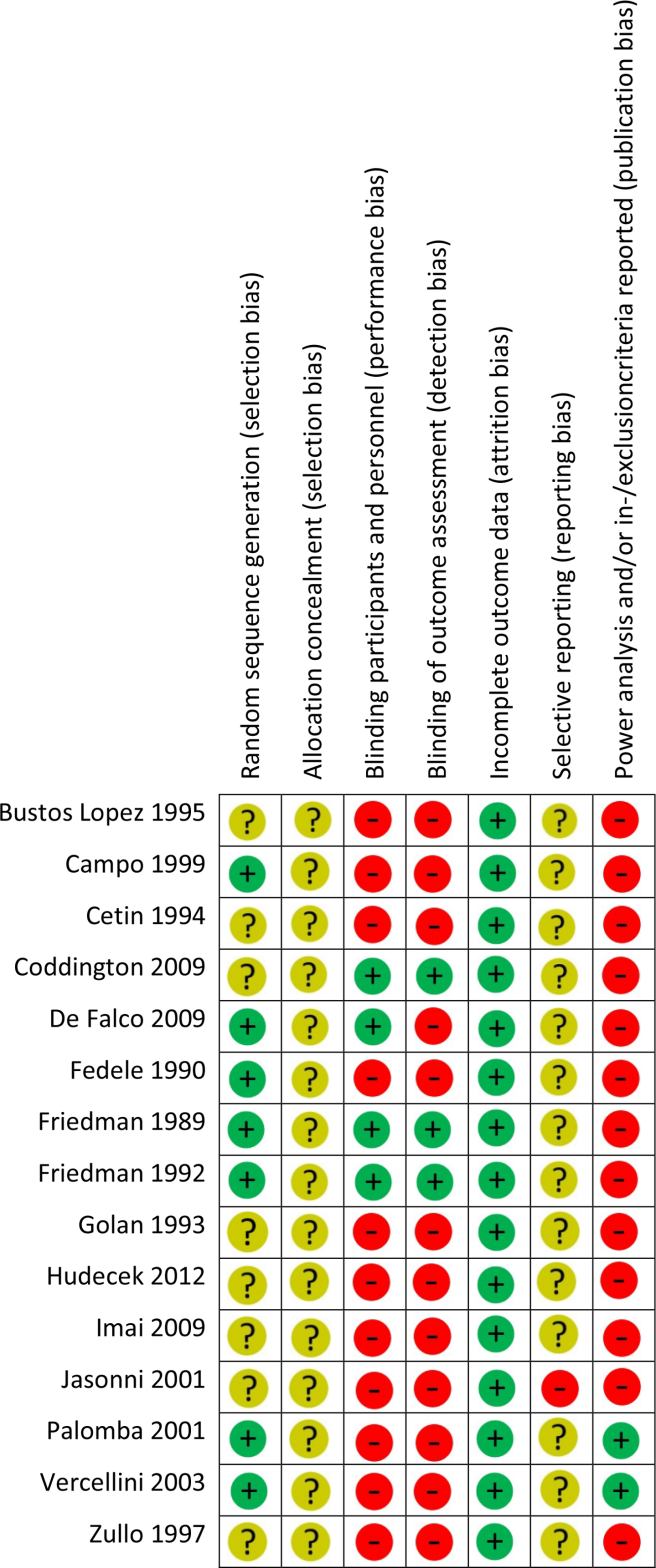
Risk of bias summary randomized controlled trials (RCT’s). The Cochrane Collaboration’s ‘Risk of Bias’ tool was used as described in the Cochrane Handbook. Studies were classified as low risk of bias (green circle with ‘plus’ sign), unclear risk of bias (yellow circle with ‘question mark’ sign) or high risk of bias (red circle with ‘minus’ sign).

For the included randomized clinical trials, the overall quality of the included studies was considered moderate (Figs 2 and 3). None of the studies reported clearly on allocation concealment which increases risk for selection bias. This was marked as an unclear risk. In only four of 15 included randomized trials, participants and personnel were blinded for allocation of pre-treatment. For this reason, possible performance bias cannot be excluded. None of the included studies published (a link) to their study protocol. Therefore, the risk for reporting bias is unclear. We added the item ‘publication bias’ to the quality assessment checklist, since we considered that a power analysis and the reporting of in-/exclusion criteria are essential items in a randomized trial. Unfortunately, only two studies reported on both items. Especially power analysis were frequently missed in the included studies. As a consequence, the findings were indicated as high risk of publication bias.

**Fig 4:**
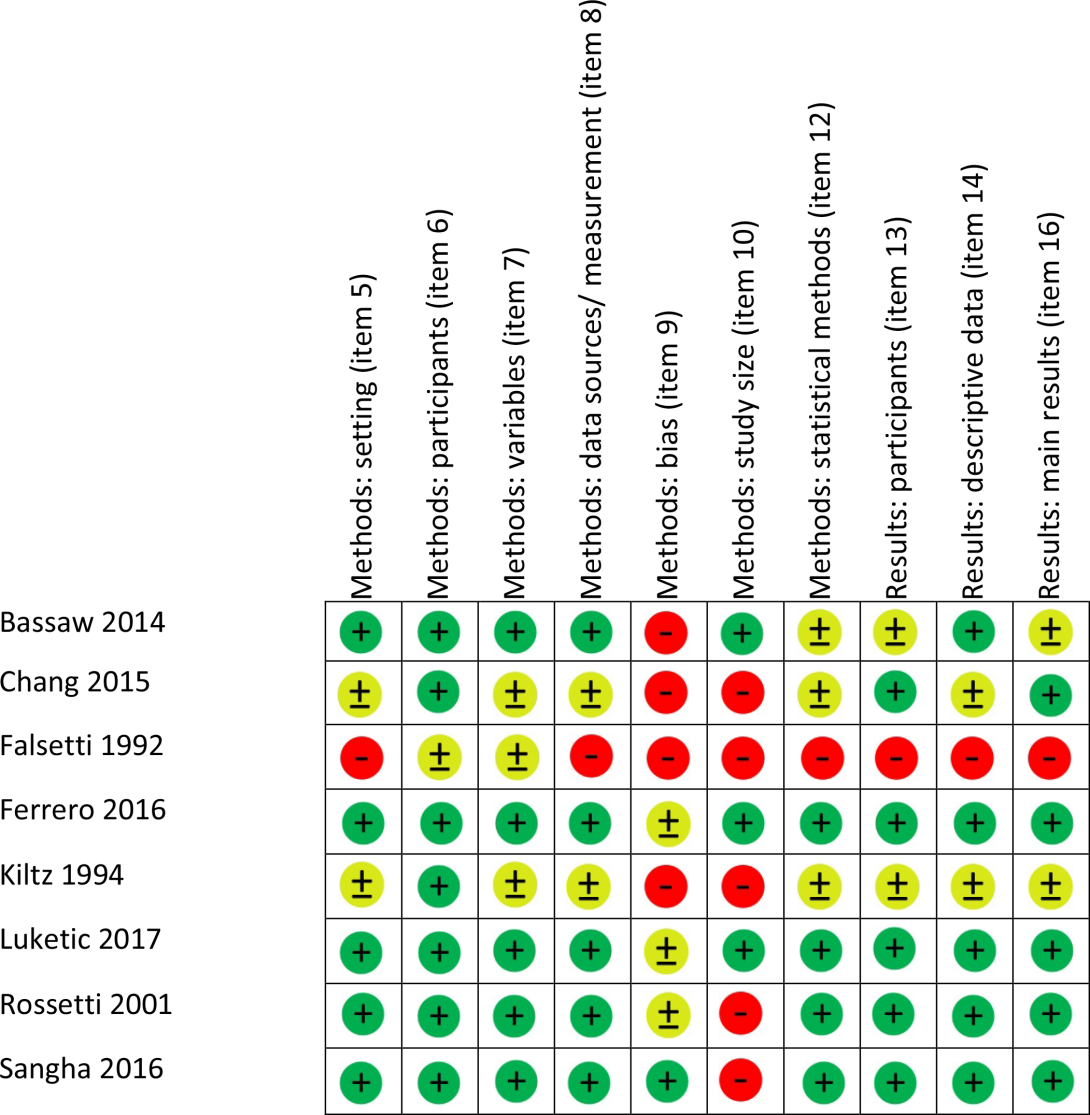
Risk of bias for included cohort studies. The ‘Strengthening the Reporting of Observational Studies in Epidemiology’ (STROBE) checklist was used to assess the risk of bias for the included cohort studies. The items checked are: setting (item 5), participants (item 6), variables (item 7), data sources/ measurements (item 8), bias (item 9), study size/ power analysis (item 10) and statistical methods (item 12). For the ‘results’ section the following items were selected: participants (item 13), descriptive data (item 14), main results (item 16). Each item was scored as low (+), moderate (±) or high (-) risk of bias.

The overall quality of the included cohort studies was considered moderate to good (Fig 4). Only one study did not score ‘low risk of bias’ on any of the selected items. The overall quality of this paper was marked as poor. Two items (bias (9) and study size/power analysis (10)) were reported very limited by the included studies. In general, the more recent studies reported a better overall quality.

Based on a funnel plot prepared for several outcomes (duration of surgery, intra-operative blood loss, frequency of blood transfusions and complication rate), there is no indication for publication bias.

### Effect of GnRHa before laparotomic myomectomy

Fifteen studies reported on laparotomic myomectomies. All studies used GnRHa as a pre-treatment. Twelve studies were randomized controlled trials of which three studies compared GnRHa and placebo and nine studies compared GnRHa and immediate surgery. In three studies buserelin or nafarelin was administered daily by nasal spray. Other studies used goserelin, leuprorelin or triptorelin given by intramuscular or subcutaneous depot injection. Detailed information for each included study on the different GnRHa preparations, their routes of administration or duration of treatment can be found in Table 1. In two studies in the laparotomic group, comparisons were made between leuprolin 3.75mg, leuprolin 7.5mg and no pre-treatment [28] and between goserelin 3.6mg two doses every four weeks, goserelin 3.6mg three doses every four weeks and no pre-treatment [26]. We chose to use the data from the leuprolin 3.75 mg and two doses goserelin 3.6mg every four weeks, since these are more comparable to the other pre-treatment schedules in our analysis. In the trials comparing pre-operative treatment with no pre-treatment, surgery was planned immediately or as soon as possible. For the outcomes recovery time and quality of life, no data were provided in the included studies.

#### Intra-operative assessment: Duration of surgery (Fig 5)

Nine studies (n = 489) reported on duration of surgery. For both randomized trials and cohort studies there was no difference found between pre-treatment or not (WMD 0.37 minutes, 95% CI -3.46 to 4.21). Heterogeneity for all studies combined was 29%.

**Fig 5:**
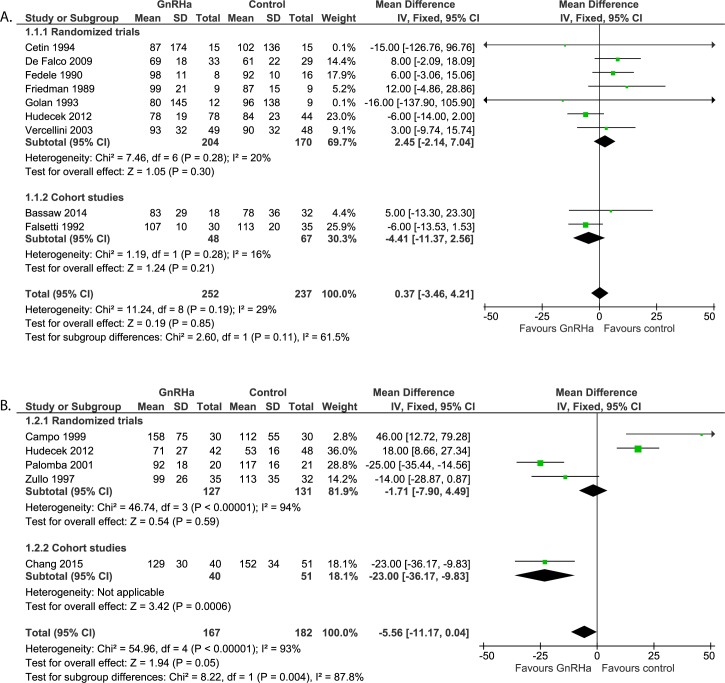
**Effect of GnRHa versus no pre-treatment before laparotomic (a) and laparoscopic (b) myomectomies.** Forest plots for meta-analysis performed on duration of surgery.

#### Intra-operative assessment: Duration of enucleation (Fig 6)

Only two studies (n = 115) registered the duration of enucleation separately from total duration of surgery. Both randomized trials reported results tending towards shorter enucleation time when no pre-treatment was used. However, overall effect was not significant (WMD 6.51 minutes, 95% CI -1.99 to 15.00; p = 0.13).

**Fig 6:**
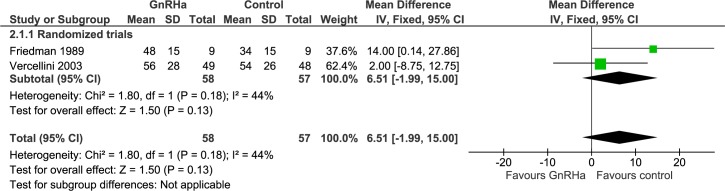
Effect of GnRHa versus no pre-treatment before laparotomic myomectomies. Forest plots for meta-analysis performed on duration of enucleation.

#### Intra-operative assessment: Blood loss (Fig 7)

Twelve studies reported on intra-operative blood loss. Eleven studies compared GnRHa with no pre-treatment or placebo (n = 536) and were used for meta-analysis. Significantly less blood loss was found in both randomized trials and cohort studies in pre-treated patients (p<0.00001). Overall, a weighted mean difference was found of -97.39 ml (95% CI -111.80 to -82.97). Two of these studies, both randomized trials, used vasoconstrictive medication [22] or an elastic tourniquet combined with vascular clamps [19] to reduce peri-operative blood loss. When eliminating these studies from statistical analysis, difference in blood loss increases slightly to -100.68ml (95% CI -115.51 to -85.86; p<0.00001) in favor of the pre-treated group. The twelfth study reporting on intra-operative blood loss by Jasonni et al. [24], evaluated the effect of six monthly depot injections of leuproreline with two monthly depot injections (n = 36). The authors did not find a significant difference in intra-operative blood loss between these two different dose groups (315±416ml vs. 336±352ml).

**Fig 7:**
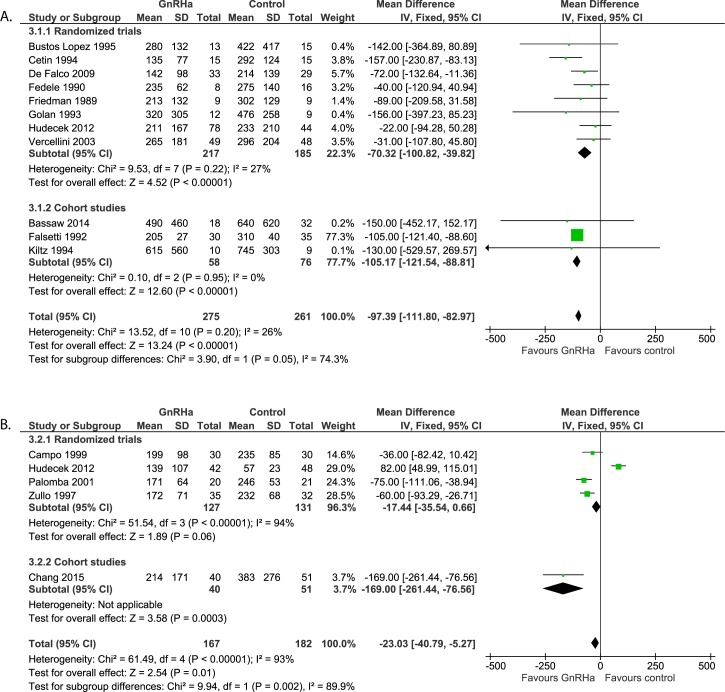
**Effect of GnRHa versus no pre-treatment before laparotomic (a) and laparoscopic (b) myomectomies.** Forest plots for meta-analysis performed on intra-operative blood loss.

#### Intra-operative assessment: Degree of difficulty of surgery

Only two studies reported on difficulty of the surgery (n = 147). A cohort study by Bassaw et al. [26] assessed difficulty in enucleating the fibroids by asking surgeon whether the enucleation was considered difficult or not. No definition of ‘difficult’ was given. Surgeons were blinded with respect to treatment group. In 11% of patients (2/18) who received GnRHa as a pre-treatment the enucleation was marked difficult due to an ill-defined capsule. In none of the patients without pre-treatment, enucleation was considered difficult. We do not know whether baseline characteristics (before pre-treatment) were similar in this group or that in particular women with large fibroids received pre-treatment.

In the randomized trial performed by Vercellini et al. [25] no differences were found in the difficulty of surgery assessed by a three point scale (easier than usual, usual or more difficult than usual). 15% of the surgeries (7/48) in the control group and 16% of surgeries (8/49) in the GnRHa group were considered more difficult than usual. However, surgeons were not blinded for treatment allocation.

#### Intra-operative assessment: Identification of cleavage planes

Three studies assessed difficulty of identification of cleavage planes (n = 224). A randomized trial by De Falco et al. [17] asked surgeons at the end of the intervention to state if they were able to promptly identify the cleavage planes between the fibroid and the surrounding myometrium or not. Surgeons were blinded to medical pre-treatment. They stated that the cleavage plane was clearly identifiable in 86.2% (25/29) of the untreated patients and in 42.4% (14/33) of GnRHa treated patients (no p-value available). A cohort study by Falsetti et al. [27] noticed that in 40% (12/30) of the GnRHa pre-treated patients the cleavage plane was more difficult to identify, they considered this related to hyaline degeneration and a smaller development of pseudo-capsula. However, surgeons were not blinded with respect to pre-treatment group. No information was provided on the method of assessment and identification of the cleavage plane in the control group. In addition, we do not know whether in particular large fibroids were pre-treated before this surgical procedure. In a randomized study performed by Vercellini et al. [25] no differences were reported in the identified cleavage planes by surgeons, who were not blinded for treatment allocation. In 10% of both treatment groups (GnRHa 5/49, control 5/48) surgeons stated they had difficulties identifying cleavage planes. As mentioned above, Bassaw et al. (14) reported that in 11% of the patients pre-treated with GnRH the enucleation was marked difficult due to an ill-defined capsule.

#### Intra-operative assessment: Proportion of vertical incisions (Fig 8)

Four studies reported on the type of incision (n = 128), all were randomized trials. In the study performed by Friedman et al. [19, 20], data was not split for GnRHa and control group. For this reason, this study could not be used in the meta-analysis. In two studies [16, 17], the proportion of vertical incisions was zero in both treatment groups. Therefore, no odds ratio could be calculated. In the remaining randomized trial reporting on this subject, the proportion of vertical incisions in the GnRHa group was 0/13 and in the control group 5/15 (OR 0.07, 95% CI 0.00 to 1.43; p = 0.08)[14].

Sensitivity analysis replacing zero events by one event in the GnRHa group results in a OR of 0.54 (95% CI 0.14 to 2.06; p = 0.36). When replacing zero events by one event in the control group, OR is 0.15 (95% 0.03 to 0.91; p = 0.04).

**Fig 8:**
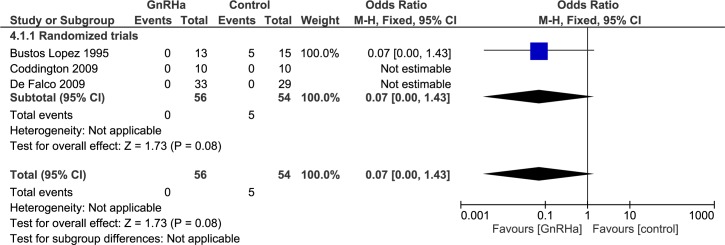
Effect of GnRHa versus no pre-treatment before laparotomic myomectomies. Forest plots for meta-analysis performed on proportion of vertical incisions.

#### Post-operative assessments: Frequency of blood transfusions (Fig 9)

Five studies (n = 205) reported on intra-operative and post-operative blood transfusions. No statistical differences were found between both groups (OR 0.84, 95% CI 0.33 to 2.11). It should be noted that the randomized trial by Vercellini et al. [25] reported no blood transfusions in both GnRHa group or no pre-treatment group and therefore no OR could be calculated. Sensitivity analysis replacing zero events by one event for both groups does not show any divergent results (OR 0.94, 95% CI 0.39 to 2.25 in case of one event in the GnRHa group and OR 0.77, 95% CI 0.32 to 1.86 in case of 1 event in the control group).

**Fig 9:**
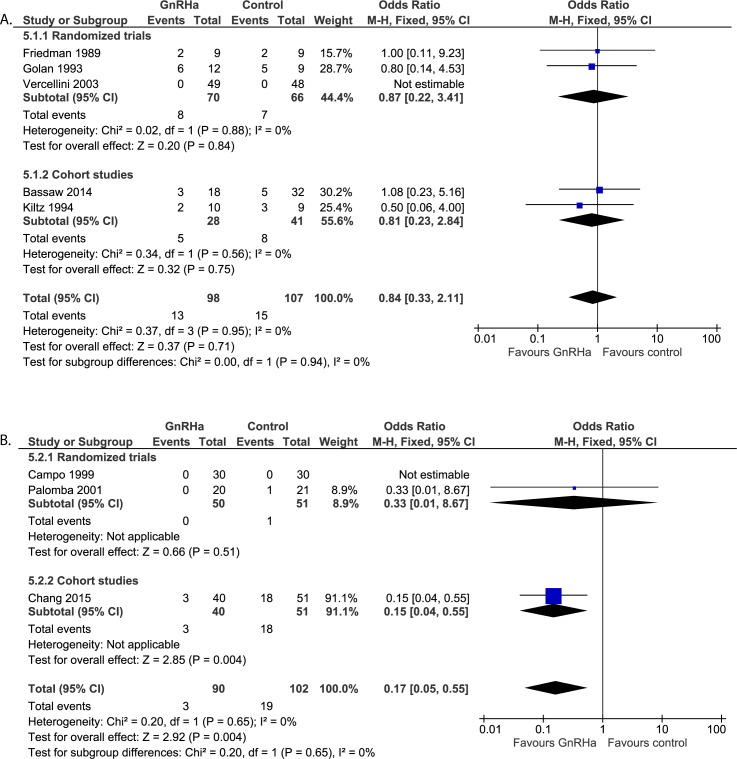
**Effect of GnRHa versus no pre-treatment before laparotomic (a) and laparoscopic (b) myomectomies.** Forest plots for meta-analysis performed on frequency of blood transfusions.

#### Post-operative assessments: Complication rate (Fig 10)

Six studies reported on complication rate post-operatively (n = 332). The study performed by Hudecek et al. [22] could not be used for meta-analysis, since no complications occurred in both treatment groups, so no odds ratio could be calculated. The following complications were reported: post-operative fever (GnRHa 19/174; control 22/158), wound infection (GnRHa 1/174; control 0/158), re-operation due to expanding subfascial hematoma (GnRHa 0/174; control 1/158), wound hematoma (GnRHa 0/174; control 2/158) and stress urine incontinence (GnRHa 1/174; control 0/158). No differences were found in the overall complication rates between both treatment groups (OR 0.83, 95% CI 0.41 to 1.67). Performing sensitivity analysis by replacing zero events by one event in both treatment for the study by Hudecek et al. does not affect the results (OR 0.86, 95% CI 0.44 to 1.69 in case of 1 event in the GnRHa group and OR 0.76, 95% CI 0.39 to 1.51 in case of 1 event in the control group.

**Fig 10:**
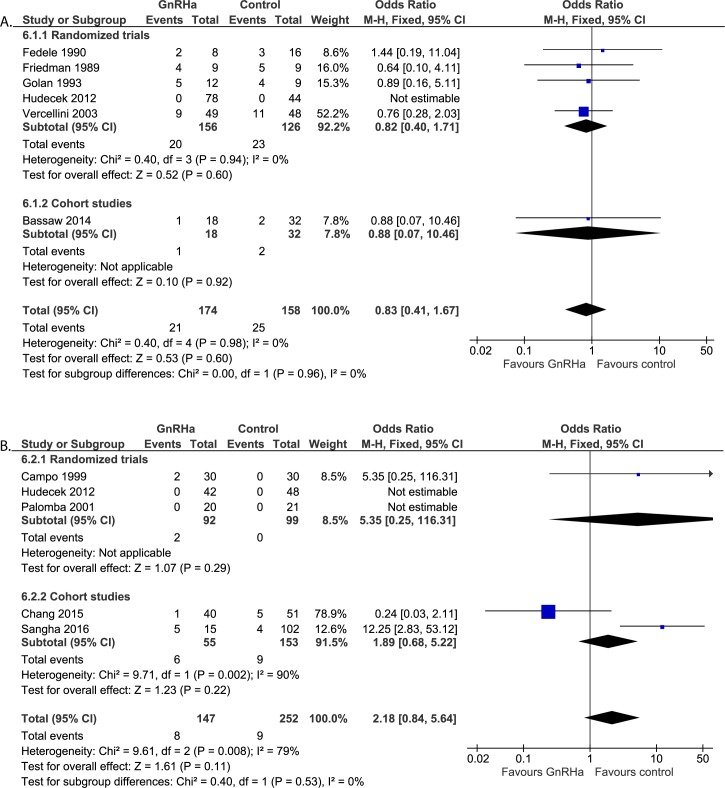
**Effect of GnRHa versus no pre-treatment before laparotomic (a) and laparoscopic (b) myomectomies.** Forest plots for meta-analysis performed on complication rate.

#### Post-operative assessments: Duration of hospital stay (Fig 11)

Five studies reported on duration of hospital stay after laparotomic myomectomy. One study [21] did not report SD, ranges or SEM and was excluded for meta-analysis. The remaining four studies (n = 287) did not report a difference between both groups (WMD 0.02 days, 95% CI -0.20 to 0.25).

**Fig 11:**
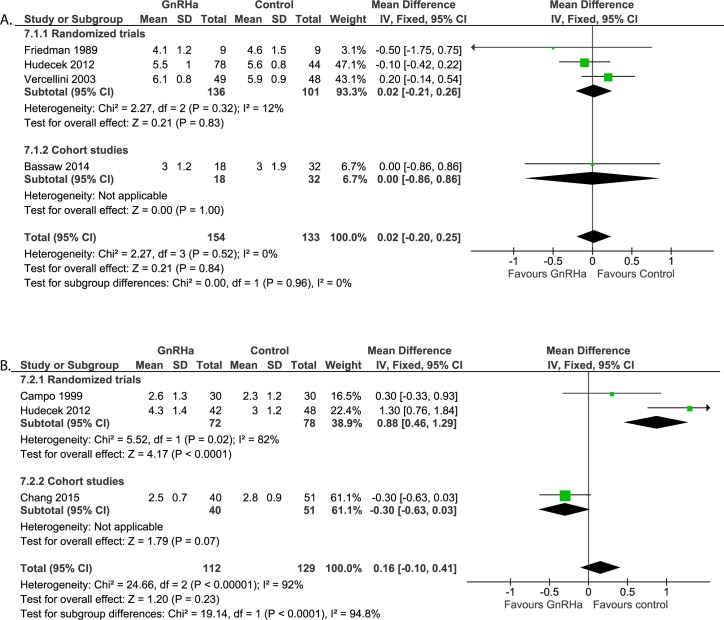
**Effect of GnRHa versus no pre-treatment before laparotomic (a) and laparoscopic (b) myomectomies.** Forest plots for meta-analysis performed on duration of hospital stay.

#### Post-operative assessments: Frequency of recurrence of fibroids (Fig 12)

Four randomized trials reported on recurrence of fibroids after open myomectomy (n = 252). The follow-up period varied from 8 weeks to 38 months. Hudecek et al. [22] evaluated the recurrence of fibroids by second look laparoscopy 8 to 12 weeks after the first surgery. It is unclear whether physicians performing the laparoscopy were blinded. Sizes of the recurrent fibroids were not reported. In addition, it was not reported if and how the existence of eventual small intramural or submucosal fibroids were evaluated. The other three studies used transvaginal ultrasound to evaluate recurrent rate. Fedele et al. [18] reported only small fibroids of <1.5cm at six months follow-up. The physician performing the ultrasound was unaware of treatment allocation. The double blind randomized trial by Friedman et al. [20], performed follow-up ultrasound at 27 and 38 months after surgery and reported recurrent fibroids in 11 of 18 patients (61%) varying from 1 to 5.6cm. The unblinded randomized trial by Vercellini et al. [25] did not report on the size of the recurrent fibroids at six monthts follow-up. The overall OR for recurrence rate of fibroids for these studies is 0.82 (95% CI 0.41 to 1.63) which is not statistically significant. Heterogeneity between the studies is large (82%). When eliminating the study performed by Hudecek et al. [22], heterogeneity decreases to 0%. The risk for recurrence is significant higher after pre-treatment with GnRHa compared to no pre-treatment or placebo (OR 3.98, 95% CI 1.26 to 12.59; p = 0.02).

**Fig 12:**
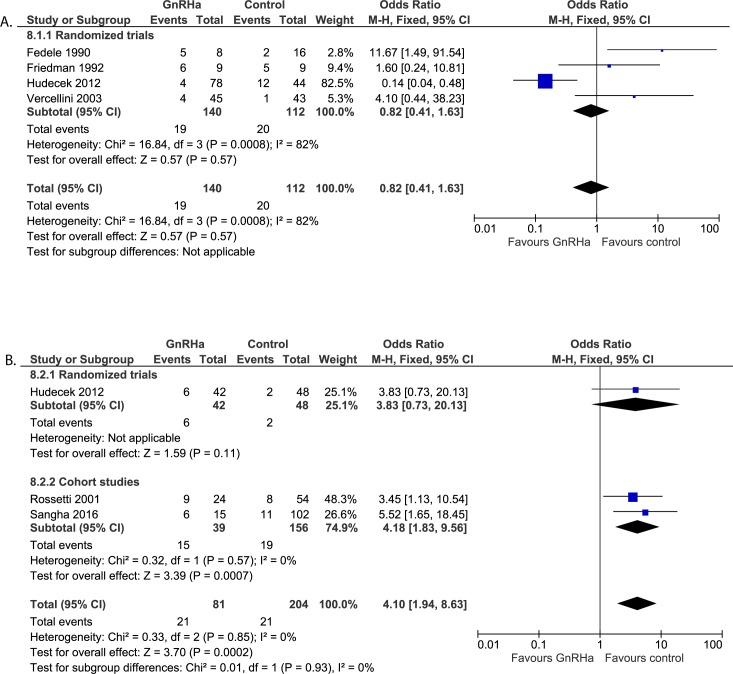
**Effect of GnRHa versus no pre-treatment before laparotomic (a) and laparoscopic (b) myomectomies.** Forest plots for meta-analysis performed on recurrence of fibroids.

#### Post-operative assessments: Frequency of uterine adhesions

Two randomized trials reported on prevention of uterine adhesions after pre-operative treatment with GnRHa (n = 35). In the study performed by Coddington et al. [16], patients underwent an initial laparotomic myomectomy followed by second look laparoscopy two to ten weeks later to evaluate and measure the adhesions present. Surgeons were blinded for treatment allocation. They found no difference in adhesions formation compared with placebo. Patients in the GnRHa treatment group of the study by Imai et al. [23] also received GnRHa for four weeks after their initial laparotomic myomectomy. Second look laparoscopy was performed 16 months after the initial surgery. A statistically significant reduction was seen in adhesion formation after the combined pre- and post-operative medical treatment with GnRHa in comparison to no pre-treatment (adhesion scores (0 to 3): 0.2±0.4 vs. 2.0±1.0; p<0.0001).

#### Effect of GnRHa before laparoscopic myomectomy

Seven studies reported on GnRHa and laparoscopic myomectomies (Table 2). Four randomized controlled trials and three cohort studies comparing GnRHa to no pre-treatment. No studies compared GnRHa to placebo. The used GnRHa preparations included triptorelin, leuprorelin or goserelin by intramuscular or subcutaneous depot injection (Table 2). One RCT (three arms) also assessed the effect of the combined use of GnRHa with tibolone before laparoscopic myomectomy [30] by randomizing patients into either leuproreline 3.75mg plus tibolone, leuproreline 3.75mg plus placebo or no pre-treatment. The group using leuproreline 3.75mg plus placebo was used for the statistical analysis in this review, given its similarity to most other studies. In the trials comparing pre-operative treatment with no pre-treatment, surgery in the no pre-treatment group was planned immediately or as soon as possible. For the outcomes duration of enucleation, degree of difficulty of surgery, identification of cleavage planes, frequency of uterine adhesions, recovery time and quality of life, no data were provided in the included studies.

#### Intra-operative assessment: Duration of surgery (Fig 5)

Five studies (n = 349) reported on duration of surgery after pre-treatment with GnRHa versus no pre-treatment. Overall, there is no statistically significant difference in surgery time between pre-treatment with GnRHa compared to no pre-treatment (WMD -5.56 minutes, 95% CI -11.17 to 0.04; p = 0.05). Four studies are randomized trials with a heterogeneity of 94% (WMD -1.71 minutes, 95% CI -7.90 to 4.49). One prospective cohort study [32] showed a significant reduction in duration of surgery of 23 minutes (95% CI -36.17 to -9.83) when pre-treated with GnRHa (p = 0.0006).

#### Intra-operative assessment: Blood loss (Fig 7)

Five studies reported on intra-operative blood loss (n = 349). All of these studies used vasoconstrictive medication pre-operatively to reduce blood loss in both groups. Blood loss is significant reduced when pre-treated with GnRHa compared to no pre-treatment (WMD -23.03ml, 95% CI -40.79 to -5.27; p = 0.01). It should be noted that heterogeneity between the four randomized trials is large (I^2^ = 94%) this is mainly due to the study performed by Hudecek et al. [22]. When excluding this study from analysis, the overall positive effect in the reduction of blood loss of GnRHa pre-treatment increases from 23.03ml to 65.84ml (95% CI -86.91 to -44.77; p<0.0001) and heterogeneity reduces to 0% in the randomized trials and to 55% overall.

#### Intra-operative assessment: Conversion rate

Only the study of Sangha et al. [36] did not report on conversion rate. In all other studies performing laparoscopic myomectomy (n = 430), no conversions to open procedures occurred.

#### Post-operative assessments: Frequency of blood transfusions (Fig 9)

Only three studies reported on the frequency of post-operative blood transfusions (n = 192). However, meta-analysis could only be performed on two of three studies, since none of the patients in the study performed by Campo et al. [29] required a blood transfusion (n = 60). Therefore, the odds ratio could not be calculated. A reduced rate for blood transfusions was found in patients pre-treated with GnRHa compared to immediate surgery (OR 0.17, 95% CI 0.05 to 0.55; p = 0.004). A sensitivity analysis replacing zero events by one event for the study by Campo et al. does not adversely affect the results (OR 0.25, 95% CI 0.09 to 0.70; p = 0.008 in case of 1 event in the GnRHa group and OR 0.18, 95% CI 0.06 to 0.55; p = 0.003 in case of 1 event in the control group).

#### Post-operative assessments: Complications (Fig 10)

Five studies reported on post-operative complication rate (n = 399). However, no odds ration could be calculated from the study performed by Hudecek et al. [22] and by Palomba et al. [30] since no complications occurred in both treatment groups (n = 90 and n = 41 respectively). The following complications were reported in the remaining studies: post-operative fever (GnRHa 2/147, control 0/252), post-operative hematoma (GnRHa 1/147, control 5/252) and re-operation (GnRHa 5/147, control 4/252). No statistical difference was found between the two treatment groups (OR 2.18, 95% CI 0.84 to 5.64). Heterogeneity between studies was large (I^2^ = 79%). When performing sensitivity analysis by replacing zero events by one event in both treatment groups for the studies by Hudecek and Palomba et al., no statistical difference is found either (OR 2.36, 95% CI 0.98 to 5.65 in case of 1 event in the GnRHa group and OR 1.56, 95% CI 0.67 to 3.63 in case of 1 event in the control group).

#### Post-operative assessments: Duration of hospital stay (Fig 11)

Three studies reported on the duration of hospital stay (n = 140). Overall, no statistical difference was found between both treatment groups (WMD 0.16 days, 95% CI -0.10 to 0.41). It should be noted that heterogeneity between the studies is large (I^2^ = 92%).

#### Post-operative assessments: Frequency of recurrence of fibroids (Fig 12)

Three studies reported on recurrence rate of fibroids (n = 285). A significantly higher recurrence rate was found in patients who were pre-treated with GnRHa (OR 4.10, 95% CI 1.94 to 8.63). This difference was only found in both cohort studies and not in the randomized trial reporting on recurrence rate, so we do not know whether selection for pre-treatment may have played a roll. The follow-up period in this trial was only eight to twelve weeks after primary surgery [22]. Rossetti et al. [35] and Sangha et al. [36] performed transvaginal ultrasound to assess recurrence rate till 40 months and 48 months respectively after primary surgery.

### Effect of ulipristal acetate before laparotomic myomectomy

No studies were found using ulipristal acetate as pre-treatment before laparotomic myomectomy.

### Effect of ulipristal acetate before laparoscopic myomectomy

**Fig 13:**
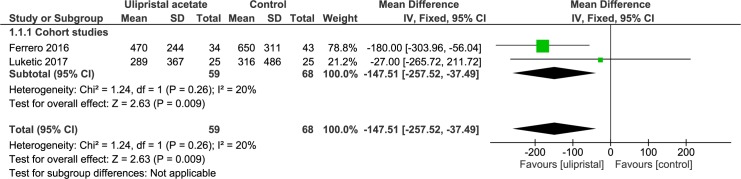
Effect of ulipristal acetate versus no pre-treatment before laparoscopic myomectomy. Forest plot for meta-analysis performed on intra-operative blood loss.

Two studies reported on ulipristal acetate before laparoscopic myomectomies [33, 34]. Both are retrospective cohort studies and compared a daily dose of 5mg of ulipristal acetate for twelve consecutive weeks to no pre-treatment (Table 2). The study by Ferrero et al. (n = 77) reported a statistically significant shorter operative time (137.6±26.8 minutes vs. 159.7±26.8 minutes; p<0.001) and significantly less post-operative blood transfusions (0/34 vs. 6/43; p = 0.031) when pre-treated with ulipristal acetate. No significant difference was found for complication rate (4/34 vs. 4/43; p = 0.726) and duration of hospital stay (2.0±1.0 days vs. 2.0±0 days; p = 0.053). No patients required conversion to laparotomy in both groups. Given the design we do not know whether a selected group of patients was pre-treated with ulipristal acetate. Both studies reported on intra-operative blood loss (n = 127) (Fig 13). Blood loss is statistically lower when pre-treated with ulipristal acetate compared to no pre-treatment (WMD -147.51ml, 95% CI -257.52 to -37.49; p = 0.009).

The study by Luketic et al. (n = 50) also compared surgical experience of laparoscopic and robotic myomectomies in women pretreated with ulipristal acetate by assessing surgical videos of 50 patients by two independent and blinded gynecologists. The non-validated assessment tool contained questions on depth of incision in the myometrium, identification of the cleavage plane, detachment of fibroid and endometrium, amount of bleeding and consistency of the fibroids. Overall, there was no difference in surgical experience for myomectomies of patients pretreated with ulipristal acetate versus no pre-treatment (global rating score 12.4 vs. 13.4; p = 0.23). There was also no difference in identification of the cleavage plane between both groups (p = 0.68).

None of the included studies reported on duration of enucleation, frequency of recurrence of fibroids, frequency of uterine adhesions, recovery time and quality of life.

## Discussion

### Main findings

This systematic review evaluated the effectiveness of medical pre-treatment with GnRHa or ulipristal acetate before laparotomic and laparoscopic myomectomies on various relevant intra-operative and post-operative outcomes. The overall quality of the included randomized trials was considered moderate and for the included cohort studies moderate to good. In laparotomic myomectomy, pre-treatment with GnRHa decreases intra-operative blood loss with almost 100ml. No difference was found in frequency of blood transfusions. Pre-treatment with GnRHa might also decrease uterine adhesions, however studies show conflicting results. All other outcomes regarding pre-treatment with GnRHa before laparotomic myomectomies did not show any significant results.

Pre-treatment with GnRHa before laparoscopic myomectomies reduces intra-operative blood loss with 23ml. It also shows significant less frequent blood transfusions and an increased risk for fibroid recurrence rate, however it should be noted that these results are mainly based on the results of cohort studies. For the other outcomes regarding pre-treatment with GnRHa before laparoscopic myomectomy, no significant results were found.

No studies were found reporting on pre-treatment with ulipristal acetate before laparotomic myomectomy. Only two retrospective cohort studies reported on pre-treatment with ulipristal acetate before laparoscopic myomectomy. These cohort studies found a significant effect for intra-operative blood loss, duration of surgery and frequency of blood transfusions.

The meta-analysis performed in this review show only marginal benefits of pre-treatment before both laparotomic and laparoscopic myomectomies on intra-operative and post-operative outcomes. For some outcomes cohort studies and randomized trials show conflicting results which might result in wrongful conclusions mainly based on results of cohort studies. Possible selection bias results in a moderate level of evidence in cohort studies. In theory it could be that in particular patients with larger or more fibroids had an indication for pre-treatment. Besides, for all studies reporting a statistically significant difference in intra-operative outcomes, clinical relevance could be discussed. Therefore, pre-treatment should not yet be offered routinely prior to myomectomy for the purpose of making surgery safer and/or easier.

### Strengths and limitations

To our knowledge, this is the first systematic review and meta-analysis published reporting on both GnRHa and ulipristal acetate before laparotomic and laparoscopic myomectomies. A Cochrane review by Lethaby at al. [4] focused on pre-treatment with GnRHa before surgery. They evaluated both hysterectomies and myomectomies and evaluated these data separately. However, no distinction was made between laparotomic and laparoscopic myomectomies. In our opinion, both surgical approaches should be evaluated as two different entities, since an effect for example on the texture or vascularity of the fibroids may be of more effect in the laparoscopic approach than in the laparotomic approach.

Another strength of this review is the methodological quality ensured by following the Prisma guidelines for systematic reviews and meta-analysis [7] and that the outcomes for randomized trials and cohort studies were reported separately since confounders may play a larger role in the cohort studies.

A possible limitation of this review is that no distinction was made between different routes of administration, different GnRHa preparations (e.g. leuprorelin, goserelin), different dosages of GnRHa and the frequency of monthly depot injections. Since these factors are so various in the included studies, it was not possible to perform different (sub)analysis. Most studies injected GnRHa intramuscularly or subcutaneously. However, in three studies GnRHa was given by nasal spray [15, 18, 23]. Although differences in pharmacological availability may occur, we did not observe clear differences in outcomes. However, this cannot be excluded. Nasal spray is self-administered once daily, this could lead to less optimal compliance and as a consequence to underestimation of the effect.

In two studies, two different GnRHa dosages were compared with a control group. Bassaw et al. [26] randomized between either two or three doses of goserelin pre-operatively. There were no significant differences between these two groups for the outcomes assessed in this review. The study by Kiltz et al. [28] compared three monthly intramuscular injections of leuprolin 3.75mg and 7.5mg. No differences were found between these groups for the outcomes assessed in this review. One study compared the effectiveness of short term administration of tibolone in patients treated with GnRHa to treatment with GnRHa and placebo or to pre-treatment with iron tablets only [30]. The authors concluded that administration of tibolone in patients treated with GnRHa before laparoscopic myomectomy does not change the effectiveness of GnRHa administered alone.

Kamath et al. [37] performed a systematic review and meta-analysis on GnRHa prior to hysteroscopic resection of submucous fibroids also evaluating duration of surgery. Two trials (n = 86) were included showing significant shorter operation time when pre-treated with GnRHa (WMD -5.34 minutes; 95% CI -7.55 to -3.12). No significant effect on duration of surgery was found in our meta-analysis. It should be noted that results in the review by Kamath et al. are based on only two small trials. Besides, clinical relevance of a shorter duration of surgery of only five minutes could also be discussed.

The results of our meta-analysis indicate that GnRHa may have a negative impact on the recurrence rate of fibroids. A possible alternative explanation could be shrinkage of small fibroids during GnRH agonist therapy which are not recognized during surgery. After discontinuing pre-treatment, these small fibroids increase in size and are therefore marked as recurrent fibroids. It should be noted that the results of this meta-analysis are mainly based on two cohort studies, so we do not know whether selection for pre-treatment may have played a role. The randomized trial on this subject does not report any significant results.

### Implications for future research

Future research should focus on high quality double-blinded randomized trials, evaluating relevant outcomes such as change in surgical approach (i.e. pfannenstiel vs. vertical incision), incision size, recovery after surgery and quality of life. Specific parameters like fibroid size, type and location should be taken into account.

Results of ulipristal acetate seem to be promising, however evidence is insufficient to support standard use in the treatment of women with uterine fibroids. For laparotomic and laparoscopic myomectomy ulipristal acetate need to be evaluated in comparison to placebo or GnRHa. A prospective pilot study by Bizzarri et al. [38] focused on pre-treatment with triptorelin, letrozole and ulipristal acetate before hysteroscopic myomectomy (n = 38). They concluded that pre-operative treatment with triptorelin and letrozole decreases the hysteroscopy time and the volume of fluid absorbed during hysteroscopic resection of fibroids. Ulipristal acetate did not show this statistically significant effect.

## Conclusion

There is high evidence from randomized trials and cohort studies that administration of GnRHa prior to laparotomic myomectomy reduces blood loss. It may also decrease uterine adhesion formation, however quality of evidence is considered moderate. Pre-treatment with GnRHa before laparoscopic myomectomy reduces blood loss, the frequency of blood transfusions and might increase recurrence rate of fibroids, however it should be taken into account that some results are mainly based on cohort studies and therefore quality of evidence is considered low. Besides, clinical relevance could be discussed for most significant results on intra-operative outcomes. Other pre-treatment agent ulipristal acetate has not been investigated sufficiently for relevant surgical outcomes and should not be prescribed routinely in order to improve surgical outcomes. Research on pre-treatment before myomectomy should focus on randomized comparisons with relevant outcomes.

## Supporting information

S1 File Search strategy for all databases. (DOCX)

S2 File PRISMA 2009 checklist. (DOC)
