# Supplementary material for: Pre-treatment with GnRHa or ulipristal acetate prior to laparoscopic and laparotomic myomectomy: A systematic review and meta-analysis
Source: PLoS One. 2017 Oct 16;12(10):e0186158. doi: 10.1371/journal.pone.0186158 (PMC5643155; doi:10.1371/journal.pone.0186158)
Supplement: S1 File — (DOCX) [file pone.0186158.s001.docx]

**Supporting Information File 1**

**Search strategy for PubMed (3 April 2017)**

[Mesh] = Medical subject headings (MeSH)

[Mesh:NoExp] = MeSH, without explosion

[Supplementary Concept] = special MeSH-terms for substance names

[tiab] = words in title OR abstract

| **Search** | **Query** | **Items found** |
| --- | --- | --- |
| **#6** | #1 AND (#2 OR #3 OR #4) AND #5 | **819** |
| **#5** | "Minimally Invasive Surgical Procedures"[Mesh:NoExp] OR "Laparoscopy"[Mesh:NoExp] OR laparoscop*[tiab] OR "surgery" [Subheading] OR "Surgical Procedures, Operative"[Mesh] OR "Surgeons"[Mesh] OR "Perioperative Period"[Mesh] OR "Perioperative Care"[Mesh] OR surger*[tiab] OR surgical*[tiab] OR surgeon*[tiab] OR operation*[tiab] OR operative*[tiab] OR perioperati*[tiab] OR incisi*[tiab] OR extracti*[tiab] OR excisi*[tiab] OR invasive*[tiab] OR laparotom*[tiab] OR preoperati*[tiab] OR pre operati*[tiab] OR pretreat*[tiab] | **4,661,712** |
| **#4** | "letrozole" [Supplementary Concept] OR "Triazoles"[Mesh] OR Femara[tiab] OR cgs 20267[tiab] | **30,998** |
| **#3** | "Gonadotropin-Releasing Hormone"[Mesh] OR gonadotropin releasing hormone*[tiab] OR luteinizing hormone releasing hormone*[tiab] OR gnrh*[tiab] OR gonadoliberin*[tiab] OR gonadorelin*[tiab] OR lfrh*[tiab] OR lh fsh releasing hormone*[tiab] OR lh releasing hormone*[tiab] OR lh rh*[tiab] OR lhfsh releasing hormone*[tiab] OR lhfshrh*[tiab] OR lhrh*[tiab] OR luliberin*[tiab] OR fsh releasing hormone*[tiab] OR gn rh*[tiab] OR factrel[tiab] OR kryptocur*[tiab] OR cystorelin*[tiab] OR leuprorelin*[tiab] OR dirigestran*[tiab] OR enantone[tiab] OR leuprolide[tiab] OR lupron*[tiab] OR tap 144[tiab] OR tap144[tiab] OR a 43818[tiab] OR a43818[tiab] OR lucrin*[tiab] | **42,512** |
| **#2** | "ulipristal"[Supplementary Concept] OR "Mifepristone"[Mesh] OR "ulipristal acetate"[Supplementary Concept] OR "asoprisnil" [Supplementary Concept] OR "telapristone acetate" [Supplementary Concept] OR "onapristone" [Supplementary Concept] OR progesterone receptor modulat*[tiab] OR prm[tiab] OR prms[tiab] OR sprm[tiab] OR sprms[tiab] OR ulipristal*[tiab] OR progestin receptor modulat*[tiab] OR pgl4001[tiab] OR mifeprex[tiab] OR mifepriston*[tiab] OR “zk 98296”[tiab] OR zk98296[tiab] OR mifegyn*[tiab] OR “r 38486”[tiab] OR “ru 38486”[tiab] OR ru38486[tiab] OR “ru 486”[tiab] OR ru486[tiab] OR r38486[tiab] OR esmya[tiab] OR “ella norpregnadiene”[tiab] OR “j867”[tiab] OR “hrp 2000”[tiab] OR “rti 3021 022”[tiab] OR “va 2914”[tiab] OR va2914[tiab] OR “cdb 2914”[tiab] OR “rti 3021 012”[tiab] OR proellex[tiab] OR “cdb 4124”[tiab] OR cdb4124[tiab] OR “zk 98 299”[tiab] OR “zk 98299”[tiab] OR “zk 299”[tiab] OR asoprisnil*[tiab] OR lonaprisa*[tiab] OR telapriston*[tiab] OR vilaprisa*[tiab] OR zk230211[tiab] OR “zk 230211”[tiab] OR upa[tiab] | **14,877** |
| **#1** | myoma*[tiab] OR leiomyoma*[tiab] OR adenomyoma*[tiab] OR (fibroid*[tiab] AND (uterus[tiab] OR uterine[tiab])) OR fibroma*[tiab] OR fibromyoma*[tiab] OR "Leiomyoma"[Mesh] OR "Myoma"[Mesh:NoExp] OR "Uterine Myomectomy"[Mesh] OR myomectom*[tiab] | **38,722** |

**Search strategy for Embase.com (3 April 2017)**

/exp = EMtree keyword with explosion

:ab,ti = words in title or abstract

NEAR/x = words near to each other, x places apart

NEXT/x = words next to each other, x places apart

| **Search** | **Query** | **Items found** |
| --- | --- | --- |
| **#7** | **#1** AND **#5** AND **#6** | **1,499** |
| **#6** | **#2** OR **#3** OR **#4** | **132,782** |
| **#5** | **'minimally invasive procedure'**/exp OR **'laparoscopy'**/exp OR **'surgery'**/exp OR **'surgeon'**/exp OR **laparoscop***:ab,ti OR **surger***:ab,ti OR **surgical***:ab,ti OR **surgeon***:ab,ti OR **operation***:ab,ti OR **operative***:ab,ti OR **perioperati***:ab,ti OR **incisi***:ab,ti OR **extracti***:ab,ti OR **excisi***:ab,ti OR **invasive***:ab,ti OR **laparotom***:ab,ti OR **preoperati***:ab,ti OR (**pre** NEXT/1 **operati***):ab,ti OR **pretreat***:ab,ti | **5,671,715** |
| **#4** | **'letrozole'**/exp OR **femara**:ab,ti OR **'cgs 20267'**:ab,ti | **9,211** |
| **#3** | **'gonadorelin'**/exp OR (**'gonadotropin releasing'** NEAR/3 **hormone***):ab,ti OR (**'luteinizing hormone releasing'** NEAR/3 **hormone***):ab,ti OR **gnrh***:ab,ti OR **gonadoliberin***:ab,ti OR **gonadorelin***:ab,ti OR **lfrh***:ab,ti OR (**'lh fsh releasing'** NEAR/3 **hormone***):ab,ti OR (**'lh releasing'** NEAR/3 **hormone***):ab,ti OR (**lh** NEXT/1 **rh***):ab,ti OR (**'lhfsh releasing'** NEAR/3 **hormone***):ab,ti OR **lhfshrh***:ab,ti OR **lhrh***:ab,ti OR **luliberin***:ab,ti OR (**'fsh releasing'** NEAR/3 **hormone***):ab,ti OR (**gn** NEXT/1 **rh***):ab,ti OR **factrel**:ab,ti OR **kryptocur***:ab,ti OR **cystorelin***:ab,ti OR **leuprorelin***:ab,ti OR **dirigestran***:ab,ti OR **enanton***:ab,ti OR **leuprolide**:ab,ti OR **lupron***:ab,ti OR **'tap 144'**:ab,ti OR **tap144**:ab,ti OR **'a 43818'**:ab,ti OR **a43818**:ab,ti OR **lucrin***:ab,ti | **103,454** |
| **#2** | **'ulipristal'**/exp OR **'mifepristone'**/exp OR **'asoprisnil'**/exp OR **'asoprisnil ecamate'**/exp OR **'telapristone acetate'**/exp OR **'telapristone'**/exp OR **'onapristone'**/exp OR (**'progesterone receptor'** NEAR/3 **modulat***):ab,ti OR **prm**:ab,ti OR **prms**:ab,ti OR **sprm**:ab,ti OR **sprms**:ab,ti OR **ulipristal***:ab,ti OR (**'progestin receptor'** NEAR/3 **modulat***):ab,ti OR **pgl4001**:ab,ti OR **mifeprex**:ab,ti OR **mifepriston***:ab,ti OR **'zk 98296'**:ab,ti OR **zk98296**:ab,ti OR **mifegyn***:ab,ti OR **'r 38486'**:ab,ti OR **'ru 38486'**:ab,ti OR **ru38486**:ab,ti OR **'ru 486'**:ab,ti OR **ru486**:ab,ti OR **r38486**:ab,ti OR **esmya**:ab,ti OR **'ella norpregnadiene'**:ab,ti OR **j867**:ab,ti OR **'hrp 2000'**:ab,ti OR **'rti 3021 022'**:ab,ti OR **'va 2914'**:ab,ti OR **va2914**:ab,ti OR **'cdb 2914'**:ab,ti OR **'rti 3021 012'**:ab,ti OR **proellex**:ab,ti OR **'cdb 4124'**:ab,ti OR **cdb4124**:ab,ti OR **'zk 98 299'**:ab,ti OR **'zk 98299'**:ab,ti OR **'zk 299'**:ab,ti OR **asoprisnil***:ab,ti OR **lonaprisa***:ab,ti OR **telapriston***:ab,ti OR **vilaprisa***:ab,ti OR **zk230211**:ab,ti OR **'zk 230211'**:ab,ti OR **upa**:ab,ti | **21,649** |
| **#1** | **'leiomyoma'**/exp OR **'uterus myoma'**/exp OR **'leiomyomatosis'**/exp OR **'myoma'**/exp OR **'myomectomy'**/exp OR **myoma***:ab,ti OR **leiomyoma***:ab,ti OR **adenomyoma***:ab,ti OR (**fibroid*** NEAR/3 (**uterus** OR **uterine**)):ab,ti OR **fibroma***:ab,ti OR **fibromyoma***:ab,ti OR **myomectom***:ab,ti | **51,617** |

**Search strategy for Wiley/Cochrane Library (5 April 2017)**

ti,ab,kw = words in title, abstract or keyword

| **Search** | **Query** | **Items found** |
| --- | --- | --- |
| **#1** | myoma* or leiomyoma* or adenomyoma* or (fibroid* and (uterus or uterine)) or fibroma* or fibromyoma* or myomectom*:ti,ab,kw (Word variations have been searched) | **1,446** |
| **#2** | ("progesterone receptor" and modulat*) or prm or prms or sprm or sprms or ulipristal* or ("progestin receptor" and modulat*) or pgl4001 or mifeprex or mifepriston* or "zk 98296" or zk98296 or mifegyn* or "r 38486" or "ru 38486" or ru38486 or "ru 486" or ru486 or r38486 or esmya or "ella norpregnadiene" or j867 or "hrp 2000" or rti-3021-022 or "va 2914" or va2914 or "cdb 2914" or rti-3021-012 or proellex or "cdb 4124" or cdb4124 or "zk 98 299" or "zk 98299" or "zk 299" or asoprisnil* or lonaprisa* or telapriston* or vilaprisa* or zk230211 or "zk 230211" or upa:ti,ab,kw (Word variations have been searched) | **1,047** |
| **#3** | ("gonadotropin releasing" and hormone*) or ("luteinizing hormone releasing" and hormone*) or gnrh* or gonadoliberin* or gonadorelin* or lfrh* or ("lh fsh releasing" and hormone*) or ("lh releasing" and hormone*) or (lh and rh*) or ("lhfsh releasing" and hormone*) or lhfshrh* or lhrh* or luliberin* or ("fsh releasing" and hormone*) or (gn and rh*) or factrel or kryptocur* or cystorelin* or leuprorelin* or dirigestran* or enanton* or leuprolide or lupron* or "tap 144" or tap144 or "a 43818" or a43818 or lucrin*:ti,ab,kw (Word variations have been searched) | **4,356** |
| **#4** | letrozole or femara or "cgs 20267":ti,ab,kw (Word variations have been searched) | **953** |
| **#5** | laparoscop* or surger* or surgical* or surgeon* or operation* or operative* or perioperati* or incisi* or extracti* or excisi* or invasive* or laparotom* or preoperati* or (pre and operati*) or pretreat*:ti,ab,kw (Word variations have been searched) | **179,911** |
| **#6** | #1 and (#2 or #3 or #4) and #5 | **146** |

Numbers per database: CDSR: 6; DARE: 4; CENTRAL: 135; EED: 1.
